# Supplementary material for: Chromothripsis during telomere crisis is independent of NHEJ, and consistent with a replicative origin
Source: Genome Res. 2019 May;29(5):737–49. doi: 10.1101/gr.240705.118 (PMC6499312; doi:10.1101/gr.240705.118)
Supplement: Supplemental Material [file supp_gr.240705.118_Supplemental_file_1.zip › contigs/annotated_contigs/DB46/contig.4.DB46_length_806_mean_cov_7.45285359801.docx]

**DB46_length_806_mean_cov_7.45285359801**

```
TTAACTAGGTCCCATATGGTAAATAAAATAATTGGTCATATTTCCTATACTTGGAATTTTTTTAAAGTTTAGTTTCGGAGACTTCATAG
 >chr4:102843729-102844048 + E=5e-180 p=5e-03
ACAGCCCTACTGCTTGTCTTCAGCATATATTTTAATAGGCCCAACTAGTATGAATTTTGGAATTCTGGCTTTTATTCAATATTGATGTT

CTGTTCTGTTAGAATTGAAGAAACTAGAATAAGTCACATAATTGCATTTGACTGCATGCATTATAAATTTATGTGTCAGTTTTGTGCTT

GAAACTACTTGGGAATCTCTTTCAAAAATCTGGCTATCACAGAATTT|TTCTC|ATTCATTACTCTGCCTCAGCAGAGTAGGAAACAGA
                                                >chr4:102845646-102846068 + E=5e-242
AGCAGAAAGGGTCAGGATGATGTACAGCTAGCCTCCACAGCAGTATAATCATTTGGCGGTGCGTTGGGAAGGGGAGCAGGCATCACTGG

GAAAAGCAAGATGAGTAAAATATCTGAATAGTCCTTGAACGTTTCTTACAACTCAGACACATAAACAGATTATTGCATTATCAATTTAC

ATCACCAAAAGATAAATTAGCCCTAAAATTCAACAAAATAGAGGTTTTGTAAAATGAATATAGTGTGTGATTTGTATTTCCAATTTCTC

GAATAAGCATAATGTATTATATTCATCACTCAGGTTTTTTATATCAGAAATGTTATATTCTCATAAAAACAGGAGACACACATTTTTCA

TAATGTAGACATTTGTTATCC|TTTTT|TTTTTTTTTTTTATTTATTTTTTTT|CTTCATATCTTCCCTTAATTGAATAATTAGTATTT
                      >chr6:19771154-19771184 - E=3e-03   >chr4:102846093-102846132 +
TAAA|TAATATE=1e-05
```
